# Field Performances of Rapid Diagnostic Tests Detecting Human *Plasmodium* Species: A Systematic Review and Meta-Analysis in India, 1990–2020

**DOI:** 10.3390/diagnostics11040590

**Published:** 2021-03-25

**Authors:** Loick Pradel Kojom Foko, Veena Pande, Vineeta Singh

**Affiliations:** 1 Cell Biology Laboratory and Malaria Parasite Bank, ICMR-National Institute of Malaria Research, Sector 8, Dwarka, New Delhi 110077, India; kojomloick@gmail.com; 2 Department of Biotechnology, Kumaun University, Bhimtal, Uttarakhand 263001, India; veena.biotech@gmail.com

**Keywords:** malaria, rapid diagnostic tests, field performances, systematic review, meta-analysis, India

## Abstract

Rapid diagnostic tests (RDTs) have become a mainstay of malaria diagnosis in endemic countries since their implementation in the 1990s. We conducted a 30-year systematic review and meta-analysis on malaria RDTs performance in India. Outcomes of interest were sensitivity (Se), specificity (Sp), positive/negative likelihood ratio (PLR/NLR), and diagnostic odd ratio (DOR). Among the 75 studies included, most of the studies were cross-sectional (65.3%), hospital-based (77.3%), and targeted febrile patients (90.6%). Nearly half of RDTs were designed for detecting *Plasmodium falciparum* only (47.5%) while the rest were for *P. falciparum* and *P. vivax* (11.9%), and *P. falciparum*/Pan-*Plasmodium* except for *P. knowlesi* (32.3%). When compared to light microscopy (gold standard), pooled estimates of performances were: Se = 97.0%, Sp = 96.0%, PLR = 22.4, NLR = 0.02 and DOR = 1080. In comparison to polymerase chain reaction, the RDTs showed Se = 89.0% and Sp = 99.0%. Performance outcomes (Se and Sp) were similar for RDT targeting *P. falciparum* only, but decreased for mixed and non-falciparum infections. Performances of malaria RDTs are still high India. However, there is a need for developing RDTs with regard to targeting minor malarial species, individuals carrying only mature gametocytes, and *pfhrp2*-deleted parasites.

## 1. Introduction

*Plasmodium falciparum* and *Plasmodium vivax* are the main species contributing to the malaria burden worldwide. These protozoan parasites are transmitted to humans through infecting bites of female *Anopheles* mosquitoes [1]. Malaria is an important public health concern, with ~229 million of cases and 409,000 deaths reported in 2019 globally. In the current context of malaria control and elimination, many countries are scaling up strategies to achieve elimination objectives stated by the World Health Organisation (WHO) [2]. However, these countries are facing enormous challenges, especially in the diagnosis area.

Rapid diagnostic tests (RDTs) are one of the mainstays for the detection of malaria parasites in endemic regions. RDTs rely on the immunochromatographic detection of the parasites through the targeting of either malarial antigens (Ag) or human antibodies to parasite Ag [3]. Actually, a large majority of RDTs are designed for the detection of three malarial antigens, viz., histidine rich protein 2 (HRP2), lactate dehydrogenase (LDH), and aldolase. The HRP2 protein is only yielded by *P. falciparum*, while LDH and aldolase are produced by all *Plasmodium* species [4].

Historically, the diagnosis of malaria was made using light microscopy (LM), which is still used in epidemiological surveys and clinical diagnosis [5]. Unfortunately, LM has serious limitations that hinder its utilization in endemic areas, especially those with limited resources. These methods (i) request good quality material and reagents for maintaining the quality which is challenging in field areas; (ii) are time-consuming; (iii) require highly skilled and experienced microscopists; (iv) require a continuous electricity supply; and (v) include parasite losses during the washing steps, thereby reducing the chances of detecting infections, particularly low density infections [6,7]. In the early 1990s, RDTs were developed to overcome the abovementioned LM-related drawbacks. The RDTs are easy to use, rapid (results obtained within 15–20 minutes), require few skills and no electricity [4].

India is the main malaria-burdened South East Asian (SEA) country, accounting for ~4% of disease cases and 2% of deaths in 2018 [8]. At the SEA level, India contributes to 80% morbidity and 60% mortality [8]. The country is also a big consumer of RDTs, which are an essential part of the national malaria control strategy. *P. falciparum* and *P. vivax* are the dominant species in India, with a prevalence ratio of 1:1 at the national level, but with substantial variation between regions [9,10].

In the present study, we used a systematic review and meta-analysis (SR-MA) to evaluate the field performances of malaria RDTs in India. This study is particularly important in a worrying context due to the worldwide emergence of parasites with deletions in the *pfhrp2* gene that encodes the PfHRP2 protein [11,12]. As a consequence, PfHRP2-based RDTs can miss parasites lacking the *pfhrp2* gene, thereby hindering the early diagnosis of malaria patients and increasing the risk of severe complications and deaths. To the best of the knowledge of the authors, this study is the first SR-MA addressing such a topic in the country.

## 2. Materials and Methods

The present study adhered to the Preferred Reporting Items for Systematic Reviews and Meta-Analyses (PRISMA) [13]. The PRISMA flow diagram and checklist were used to describe the study selection process and the characteristics of the SR-MA, respectively (Figure S1 and Table S2, Supplementary Materials).

### 2.1. Ethical Statement

The obtaining of an ethical clearance was not requested, as the data used in this study are retrieved from publicly available previous studies.

### 2.2. Electronic Databases

Between 17 August 2020 and 17 October 2020, four electronic databases (PubMed, Wiley Library, ResearchGate and ScienceDirect) and two search engines (Google and Google scholar) were used to search potentially relevant studies.

### 2.3. Search Strategy

Main keywords used for the search in each of the databases included: “India”, “rapid malaria antigen”, “antigen detection test”, “rapid diagnostic test”, immunochromatographic test”, “performance”, “accuracy”, “usefulness”, and the different Indian regions (Tamil Nadu, Chandigarh, Andaman and Nicobar, Assam, Andhra Pradesh, Bihar, Chhattisgarh, Daman and Diu, Delhi, Goa, Gujarat, Himachal Pradesh, Jammu and Kashmir, Jharkhand, Karnataka, Kerala, Kolkata, Lakshadweep, Maharashtra, Manipur, Nagaland, Mizoram, Madhya Pradesh, Meghalaya, Odisha, Puducherry, Rajasthan, Sikkim, Tripura, Uttarakhand, Uttar Pradesh, Punjab, Haryana and West Bengal). Boolean operators (AND, OR, XOR), truncation element (*), and filter options in the different electronic databases were used to fine-tune the search.

### 2.4. Objectives

The objectives of this SR-MA were: (1) to delineate a state-of-the-art of studies on malaria RDT performances in India, (2) to determine the field performances of RDTs as compared to standard methods based on MA of included studies, and (3) to discuss the main causes of poor performances of RDTs, identify limitations, and propose solutions.

### 2.5. Eligibility Criteria

**Figure 1:**
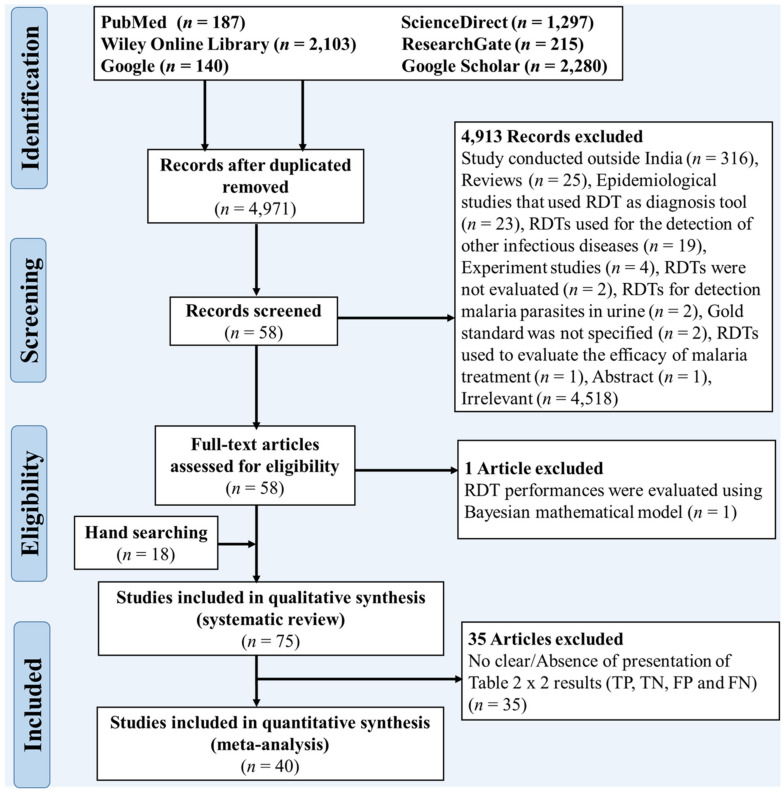
PRISMA flow diagram of the selection process of the included studies.

The studies included in the review were required (i) to be conducted in India, (ii) to be focused on the evaluation of RDT performances in the detection of malaria parasites in human blood, (iii) to be peer-reviewed and published, (iv) to be written in the English language, (v) to be published between January 1990 and September 2020 (the first RDTs have been implemented in malaria endemic areas in the early 1990s) [14,15], (vi) to use LM or molecular methods as “gold standard” method, (vii) to provide all primary data to construct 2 × 2 table of results (true positive-TP, true negative- TN, false positive-FP and false negative-FN), and (viii) the sum of TP + FN, and TN + FP are both ≥ 30 (this sample size is commonly accepted as sufficient to make a statistical analysis) [16]. Conversely, reviews, conference papers, news, and experiments studies were excluded from the study. The exhaustive list of reasons for exclusion is further presented in Figure 1 and Table S3.

### 2.6. Screening Strategy

Titles and abstracts of each publication were independently analysed. At this step, a large number of studies were directly excluded from the search. Only full texts of studies with an explicit title and/or informative abstract were retrieved. In case of the inability to retrieve the full text, the corresponding author was directly contacted by email. Papers were purchased in case of a negative reply or no reply at all from the corresponding author. The list of references of eligible studies were scrutinised to increase the chances of getting more relevant studies.

### 2.7. Data of Interest

Information of interest retrieved from each study are shown in Table 1.

### 2.8. Methodological Quality Assessment of Studies

The quality of studies included in the SR was evaluated using The Joanna Briggs Institute Critical Appraisal tools for use in JBI Systematic Reviews Checklist for Diagnostic Test Accuracy Studies available at http://joannabriggs.org/assets/docs/critical-appraisal-tools/JBI_Critical_Appraisal-Checklist_for_Diagnostic_Test_Accuracy_Studies2017.pdf (Accessed on 15 November 2020) [17,18]. This tool is based on the recommendations of the quality assessment diagnostic accuracy studies-2 (QUADAS-2) group from the university of Bristol, UK. It consists of four domains: (1) individual selection, (2) index test, (3) reference test and (4) flow and timing [17]. These domains are used to evaluate the quality of each study based on two aspects, viz., the bias risk (domains 1–4) and applicability concern (domains 1–3) [17]. We have not computed an overall quality score for each study given the absence of consensus on its calculation and interpretation [19]. The quality assessment of the studies was independently performed by two reviewers and any disagreements were resolved through discussion and consensus.

### 2.9. Data Verification for Consistency

Data of interest for SR-MA were entered in an Excel spreadsheet (Microsoft Office 2016, USA). The external control of the Excel database was performed by two external reviewers. Again, this database was also checked for consistency by two additional persons. Discrepancies were solved through discussion between all authors.

### 2.10. Outcomes to Appraise Field Performances

Parameters of interest evaluated in the present SR-MA consisted in sensitivity (Se), specificity (Sp), negative and positive likelihood ratio (NLR and PLR) and the diagnostic odds ratio (DOR). These performance parameters are defined as follows:

**Sensitivity** is the probability of a person with the disease of interest having a positive test result [20]. In this review, it was the ability of the RDT to correctly identify malaria-infected individuals.

**Specificity** is the probability of a person without the disease of interest having a negative test result [20]. In this review, it meant the ability of the RDT to correctly identify malaria-uninfected individuals.

**Positive (negative) likelihood ratio (P/NLR)** indicates how much less likely it is to find a positive (negative) test result in individuals with malaria as compared with those without a malaria infection [21]. In this study, it was how much less likely it is to find a positive (negative) RDT result in malaria-infected individuals as compared with their uninfected counterparts. Likelihood ratio values range from 0 to infinity. If the PLR is greater than 1, the more likelihood of the test being positive. In contrast, if NLR is less than 1, there is less possibility of a negative result [21].

**Diagnostic odds ratio (DOR)** of a test is the ratio of the odds of positivity in individuals with the disease relative to the odds of positivity in the individuals without the disease. DOR values range from 0 to infinity. In practice, DOR values above 1 are indicative that the test is correctly discriminating. The larger the DOR value, the more accurate the test is [22].

### 2.11. Data Management

Software including the statistical package for social science v16 for Windows (SPSS, Inc., IL, Chicago, USA) and GraphPad Prism v5.03 (GraphPad, San Diego, USA) were used to perform descriptive statistics (percentage, confidence interval at 95% and mean) and were presented as tables or charts while JASP v0.12.1 (University of Amsterdam, Netherlands) and OpenMeta Analyst (http://www.cebm.brown.edu/openmeta/doc/openMA_help.html) (Accessed on 12 October 2020) were used to perform the MA of RDT performances [23,24].

A minimum of two studies was judged sufficient to perform an MA of the parameters of interest as recommended previously [25]. The Cochrane Q test and I^2^ statistics were used to appraise the level of heterogeneity between studies included in the MA. The type of model (random or fixed effects) was chosen based on the level heterogeneity. Random effect models were preferred when I^2^ was > 75% while fixed effect models were used when I^2^ < 25% [26,27]. Arcsine transformation was used to stabilise the variance between the included studies. The sources of heterogeneity between studies were searched using subgroup analysis while the leave-one-out method (sensitivity analysis) was used to evaluate the influence of each individual study on the pooled estimates of sensitivity, specificity, PLR, NLR, and DOR [28]. Variables tested for subgroup analysis included Indian area, type of RDT (Pan only, Pf and Pv, Pf only, and Pf/Pan), the malarial Ag (HRP2, LDH, aldolase), study population (children, adults, general population), and the staining solution used for LM (Giemsa, Leishman, and Jaswant Singh–Bhattacharji).

Forest plots were used to present the results of MA while funnel plots and Egger’s test were used to detect publication bias between studies [29]. Meta-regression analysis was used to identify factors influencing the performances of RDTs. A probability value of less than 0.05 was considered statistical significant.

## 3. Results

### 3.1. Selection Process Results

Titles and abstracts of studies retrieved from electronic databases were screened for eligibility and 4914 of them were excluded as per the exclusion criteria (Figure 1).

Full texts of 58 studies were retrieved and scrutinized. Eighteen supplementary studies were identified based on the analysis of reference list of these 58 studies; thus, 76 studies were evaluated for eligibility (Figure 1). Of the 76 articles, one study was excluded, as performances of RDTs were evaluated using a bayesian statistics-based mathematical model.

The list of studies excluded from the review, along with the reasons for exclusion, is presented in Table S3. Finally, 75 articles were included in the qualitative synthesis (SR), among them 40 were eligible for the quantitative synthesis (MA).

### 3.2. Geographical Distribution of the Included Studies

**Figure 2:**
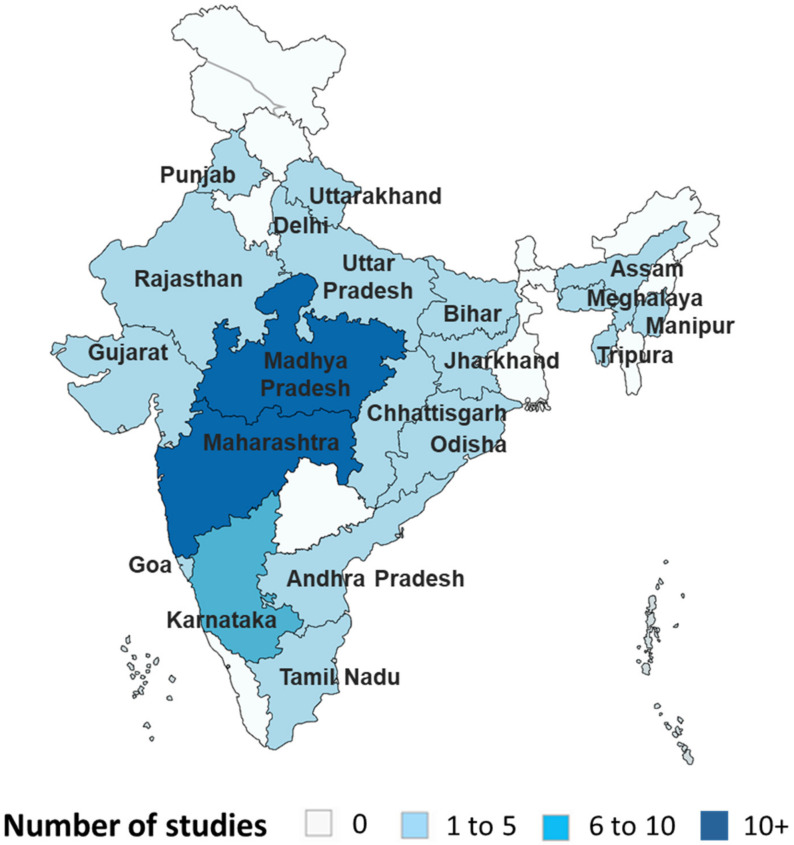
Geographical distribution of studies included in the review. The map depicted here is taken from the official website of Ministry of External Affairs, India (https://mea.gov.in/india-at-glance.htm accessed 27 October 2020).

There was a large geographical distribution of the included studies with 21 states and union territories with at least one study conducted in the included region (Figure 2). There was a high disparity in the number of studies according to the area with most of studies from three states, viz., Madhya Pradesh (16 studies), Maharashtra (11 studies) and Karnataka (7 studies). Most of the studies were from one state while the remaining two studies included several states, one in three (Assam, Meghalaya, and Manipur) and the other in six states (Assam, Bihar, Chhattisgarh, Maharashtra, Andhra Pradesh, and Tamil Nadu) (Table S4).

### 3.3. Characteristics of the Included Studies

**Figure 3:**
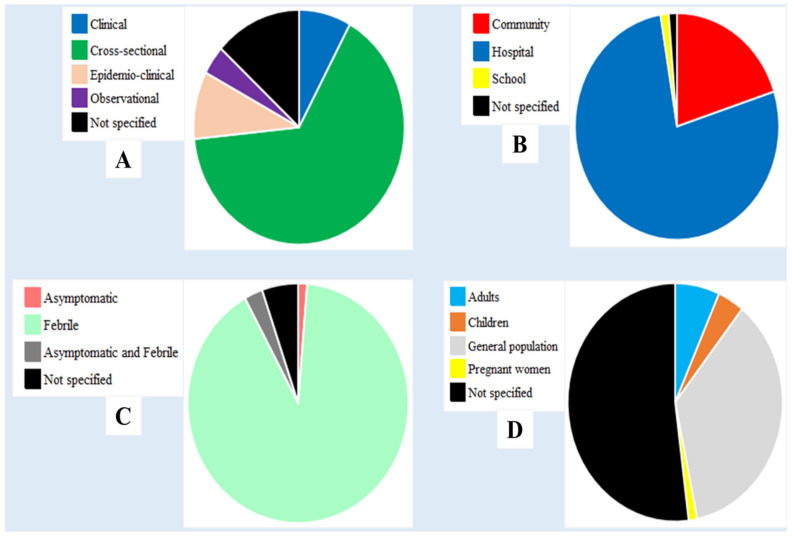
Details of the studies included in relation to design (**A**), site (**B**), clinical status (**C**), and study population (**D**).

The majority of the studies were cross-sectional (65.3%, *n* = 49), hospital-based (77.3%, *n* = 58), and recruited febrile patients (90.6%, *n* = 68) (Figure 3A,B). One study evaluated the performances of RDTs in asymptomatic patients only. Another study evaluated RDT performances in a population comprised of febrile and asymptomatic patients (Figure 3C, Table S4). Nearly one-third (30.1%, *n* = 22) of studies focused on the RDT performances in the general population while pregnant women and children were targeted by one and two studies, respectively (Figure 3D). Three studies evaluated RDT performances in patients presenting with severe malaria-suggesting signs/complications (Table S4).

### 3.4. Gold Standard Used to Evaluate the Performances of RDTs

A large proportion of studies (98.7%, *n* = 74) have used LM as a gold standard to evaluate the performances of RDTs, while molecular methods were used in 8 studies (11.1%). Staining solutions used in LM included Giemsa (62.2%, *n* = 46), Leishman (17.6%, *n* = 13) and Jaswant Singh–Bhattacharji (2.7%, *n* = 2). One study combined Leishman and Jaswant Singh–Bhattacharji, and the staining solution was not specified in the remaining studies.

### 3.5. Characteristics of RDTs Evaluated

On analysis, a total of 26 different RDT brands were evaluated across the included studies. The main RDT brands included ParaCheck Pf^®^ (12 studies), ParaSight F^®^ (10 studies), ICT Malaria Pf^™^ (10 studies), and ParaHit^®^-f (9 studies) (Table 2).

The RDTs were designed as cassette, card or dipstick. Nearly half of RDTs were specific for detection of *P. falciparum* only (47.5%, *n* = 48) while others were designed for *P. falciparum* and *P. vivax* (11.9%, *n* = 12), and *P. falciparum*/Pan-*Plasmodium* except for *P. knowlesi* (Pf/Pan (22.8%, *n* = 23), *P. falciparum*/*P. vivax*-*P. ovale*-*P. malariae* (Pf/Pvom) (5.9%, *n* = 6), and Pan only (2.9%, *n* = 3). The countries of RDT manufacturers were India, USA, Switzerland, Australia, South Korea, and Japan.

### 3.6. Methodological Quality of Included Studies

**Figure 4:**
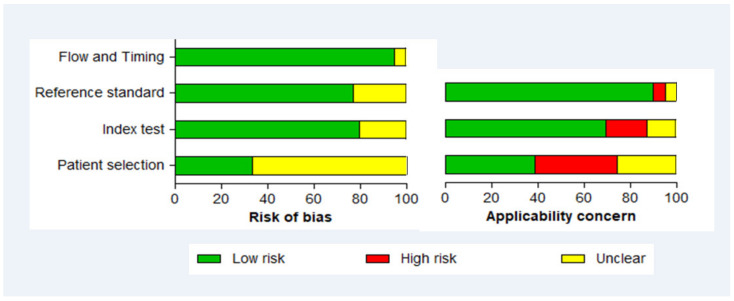
Methodological quality assessment of 40 studies included in the meta-analysis. Reviewers’ assessment of four domains (patient selection, index test, reference standard, and flow and timing) of the QUADAS-2 tool is presented in stack bars as the proportion of studies with low/high/unclear risk of bias and with low/high/unclear concerns regarding applicability [17].

A summary of the evaluation of the quality of the included studies is shown in Figure 4, and the details of this evaluation are presented in Table S5. Most of the studies showed a low risk of bias regarding index test, reference standard, and flow and timing domains. In contrast, a large proportion (66.7%) had an unclear risk of bias regarding the patient recruitment domain (Figure 4). There was a low level of concern on the applicability in relation to the “reference standard” domain, and the “index test” domain. However, the risk of concerns on the applicability regarding patient selection was high in 25.6% of the studies.

### 3.7. Detection of Plasmodium spp. Species

We selected studies that evaluated RDTs designed to detect at least two human *Plasmodium* species (i.e., HRP2 + Pan-LDH, PfLDH + Pan-LDH, HRP2 + PvLDH, and HRP2 + Panmalarial Antigen). A total of 12 studies with LM as gold standard, and 3 with molecular methods, were evaluated in this section of the review. When compared to LM, pooled estimates of performances of RDTs were: sensitivity = 97.0% (95% CI 95.0–98.0%), specificity = 96.0% (95% CI 93.0–97.0%), PLR = 22.4 (95% CI 13.6–36.9), NLR = 0.02 (95% CI 0.01–0.04), and DOR = 1080 (95% CI 413.7–2819.6). A high level of heterogeneity was found (I^2^ > 80%, *p* < 0.01).

Using PCR as the gold standard, the RDT performances were: Se = 89.0% (95% CI 23.0–100.0%) and Sp = 99.0% (95% CI 97.0–99.0%) (Figure S6). The pooled estimates of PLR, NLR and DOR were not available due to the small number of eligible studies (*n* = 3). In addition, I^2^ statistics varied between different modalities of variables included in the subgroup analysis, thereby outlining their influence on RDT performances in the detection of any plasmodial species (Table S7).

Sensitivity analysis showed no major influence of individual studies in the pooled estimates of RDTs’ performance parameters (Figure S8). Meta-regression analysis showed that DOR and sensitivity were significantly influenced by the geographical area (*p* < 0.0001 and *p* = 0.015), country of RDT manufacturer (*p*-= 0.046 and *p*-= 0.004), and LM-staining solution (*p* = 0.044 and *p-*= 0.015). Pooled specificity values were significantly influenced only by the area (*p* < 0.0001).

### 3.8. Detection of P. Falciparum

**Figure 5:**
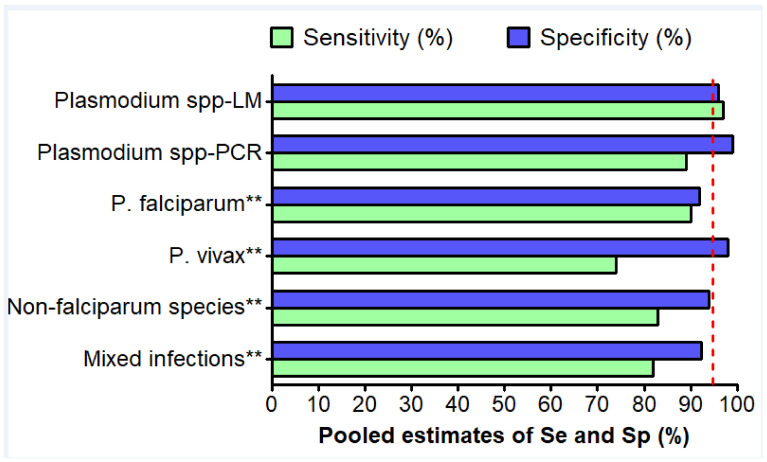
Pooled estimates of sensitivity and specificity of the RDTs against *Plasmodium* spp., *P. falciparum*, *P. vivax*, non-falciparum, and mixed infections. LM: light microscopy, PCR: polymerase chain reaction, Se: sensitivity, Sp: specificity. **LM was used as gold standard. The red line indicates the 95% threshold.

Twenty-two studies were eligible in this section. Performances of RDTs were good as pooled estimates of sensitivity and specificity were 90% (I^2^ = 92.3%; 95% CI 87.0–93.0%) and 92% (I^2^ = 92.3%; 95% CI 91.0–94.0%), respectively (Figure 5 and Figure S6).

Subgroup analysis showed the influence of some independent variables while sensitivity analysis revealed no major effect of each study on the pooled estimates of performance parameters (Table S7 and Figure S8). RDTs targeting primarily HRP2 plus another *P. falciparum* antigen were conservatively more sensitive than those targeting only HRP2 (i.e., based on the upper CI of sensitivity estimates, as presented in Figure S6). DOR, sensitivity and specificity were not significantly influenced by RDT manufacturer, LM-staining solution, and parasite antigen (Meta-regression, *p* > 0.05).

**Figure 6:**
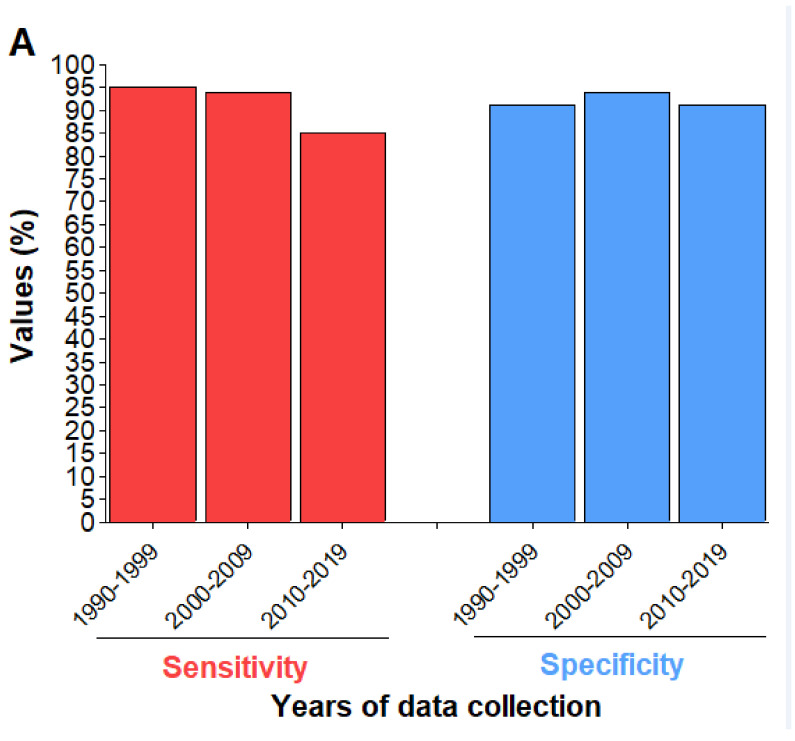

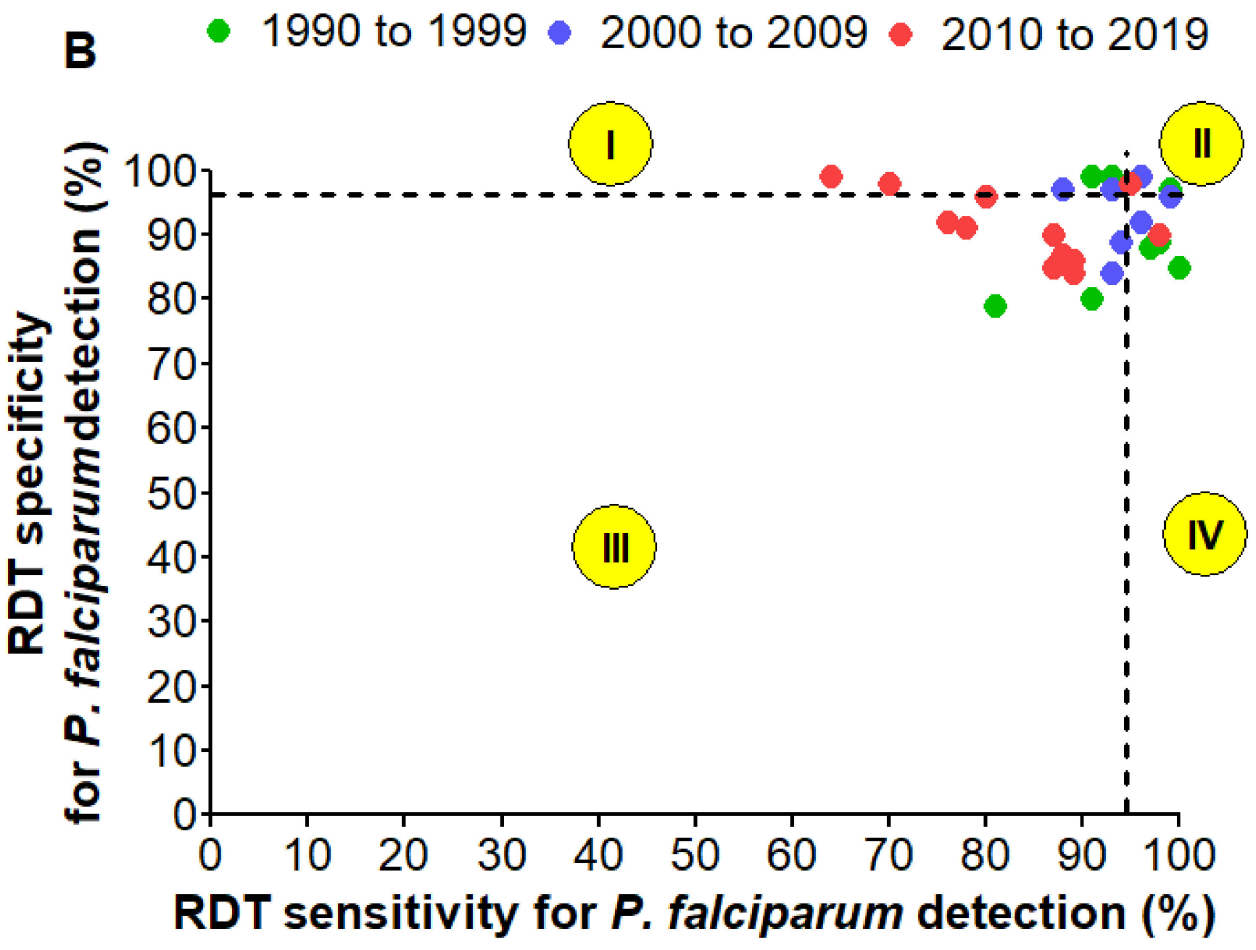
Sensitivity (Se) and specificity (Sp) of the PfHRP2-based RDTs for (**A**) the years 1990–2019, and (**B**) the individual studies (6A): The values presented are pooled estimates, (6B): A 95% threshold for Se and Sp was used to classify RDTs into four groups (I: Low Se—High Sp, II: High Se—High Sp, III: Low Se—Low Sp, and IV: High Se—Low Sp). Studies for which TP + FN < 30 and/or TN + FP < 30 were excluded.

The analysis of PfHR2-based RDT performance with regard to years of data collection revealed a slight decrease of sensitivity for these 30 years, from 95% (95% CI 91–97%, I^2^ = 87.7%; *p* = 0.001) in 1990–1999 to 85% (95% CI 91–97%, I^2^ = 87.7%; *p* = 0.001) in 2010–2019 (Figure 6A). Sensitivity and Specificity were significantly impacted by the period of data collection (Meta-regression, *p* = 0.007 for sensitivity, *p* = 0.034 for specificity). RDTs had high sensitivity and specificity in only four studies (Figure 6B).

### 3.9. Detection of P. vivax

All the ten studies eligible for this section used LM as the gold standard. Hence, no performance analysis of RDTs against PCR was possible. The performances of RDTs in the detection of *P. vivax* were lower than those reported for the detection of *P. falciparum*, especially in terms of sensitivity and DOR. The results are as follows: sensitivity = 74.0% (95% CI 64.0–82.0%), specificity = 98.0% (95% CI 97.0–99.0%), PLR = 29.5 (95% CI 19.2–45.3), NLR = 0.21 (95% CI 0.11–0.37) and DOR = 147.5 (95% CI 75.3–288.7).

Meta-regression and subgroup analysis were performed as a high level of heterogeneity between studies was found (I^2^ ≥ 74 %, *p* < 0.01) (Figure 5 and Figure S6). DOR and sensitivity were significantly influenced by the Ag targeted in the RDT (Meta-regression, *p* < 0.0001 and *p* = 0.007).

**Figure 7:**
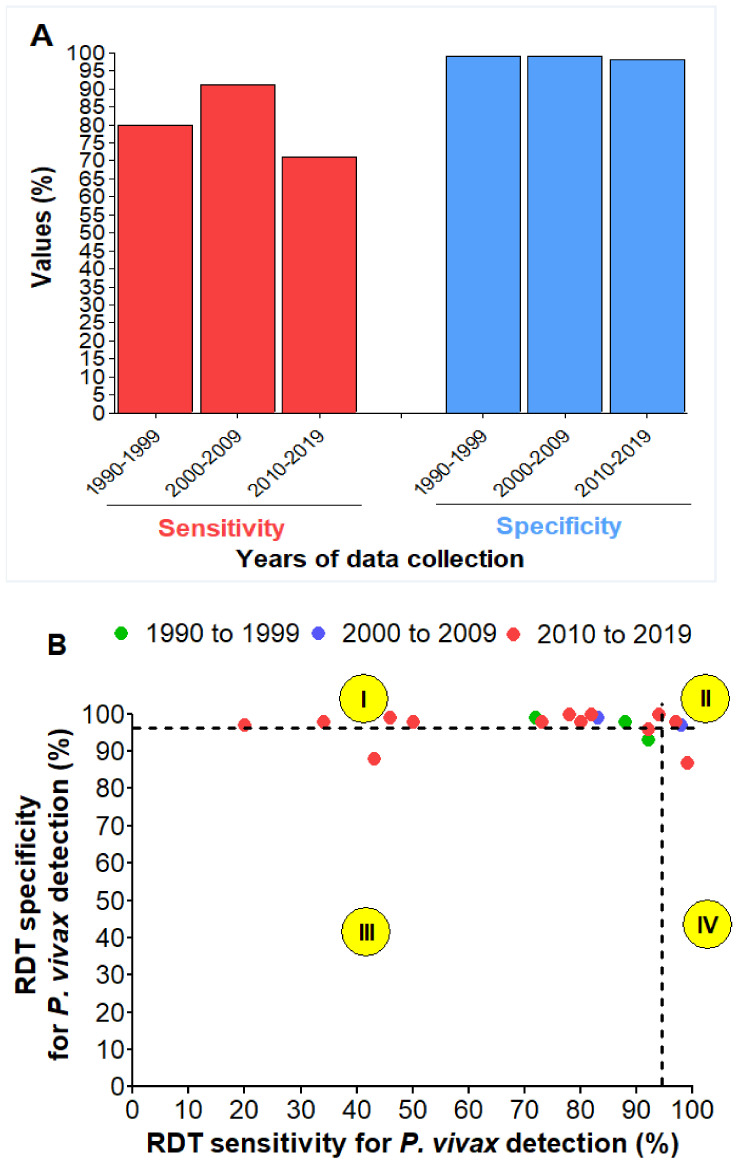
Sensitivity (Se) and specificity (Sp) of the *P. vivax*-based RDTs for (**A**) the years 1990–2019, and (**B**) the individual studies (**7A**): The values presented are pooled estimates, (**7B**): A 95% threshold for Se and Sp was used to classify RDTs into four groups (I: Low Se—High Sp, II: High Se—High Sp, III: Low Se—Low Sp, and IV: High Se—Low Sp). Studies for which TP + FN < 30 and/or TN + FP < 30 were excluded.

Similarly observed with PfHRP2-based RDTs, a decrease in sensitivity of *P. vivax*-based was observed over time (Figure 7A). Only two studies found good performances of RDTs against *P. vivax* (group II: High Se–High Sp) (Figure 7B).

### 3.10. Paired Comparison of Performances of RDT for P. falciparum and P. vivax

Only three studies compared the performances of the same RDTs between *P. falciparum* and *P. vivax*. Pooled estimates of sensitivity and specificity for the detection of *P. falciparum* were 95% (95% CI 84–98%) and 99% (95% CI 96–100%) respectively while these parameters were 95% (95% CI 77–99%) and 98% (95% CI 86–100%) for *P. vivax*. Based on the lower CI, minimum sensitivity and specificity values were 84% and 96% for *P. falciparum*, whereas they were 77% and 86% for *P. vivax*. Thus, the RDTs evaluated in these three studies were more accurate for detecting *P. falciparum* infections.

### 3.11. Detection of Non-Falciparum Species

Only one study addressed the performances of RDTs against non-falciparum species (Bharti et al. 2008 see Table S4). The authors reported sensitivity of 83% (95% CI 69–91%) and specificity of 94% (95% CI 90–96%), using LM as the gold standard (Figure 5). Another study compared the result of RDT vis-à-vis to *P. malariae*, the RDT gave positive result to only one of the 12 *P. malariae* samples identified by PCR (Haanshuus et al. 2016 see Table S4).

### 3.12. Detection of Mixed Infections

Two studies addressed the capacity of RDT to accurately detect mixed infections (Vyas et al. 2014, and Joseph and Uchila, 2018, see Table S4). The pooled estimates of sensitivity and specificity were 82% (95% CI 41.7–96.6%, I^2^ = 0%, *p* = 0.391) and 92.3% (95% CI 79.2–97.6%, I^2^ = 91.4%, *p* = 0.0001), respectively (Figure 5).

### 3.13. Gametocyte Carriage and RDT Positivity

Some studies addressed the reactivity of RDT against gametocytes. Six RDT brands (ICT Malaria Pf^™^, ParaCheck Pf^®^, ParaSight F^®^, ParaHit^®^-f, DiaMed OptiMAL^®^, and Advantage mal Card^™^) were analysed in this section.

We observed contradictory findings for PfHRP2-based RDTs only. Studies that tested ICT Malaria Pf^™^ kit found no link between RDT reactivity and presence/absence of gametocytes. In contrast, studies that evaluated ParaCheck Pf^®^, found that it was able to detect all individuals infected only with gametocytes. All the LDH-based RDTs were able to detect gametocyte infections (Table 3).

### 3.14. Intensity of RDT Test Line and Parasitemia

Results on the relation between level of parasitemia and intensity of RDT test line varied according to the RDT brand. No correlation was found with ICT Malaria Pf^™^ kit, while one study found a statistically significant positive correlation between parasitemia of either ring stage only or ring stage + gametocyte, with the test line intensity of the ParaCheck Pf^®^ RDT (Table 3).

### 3.15. RDT Performances and Level of Parasitemia

In total, 18 studies assessed the performances of RDTs according to the level of parasitemia and all of them found increased RDTs’ performances at higher levels of plasmodial parasitemia (Table S4).

### 3.16. RDT Performances against P. falciparum Isolates with Pfhrp2 Deletions

No studies included evaluated the performances of RDT in the detection of *P. falciparum* samples with deletions in the *pfhrp2* gene. We have exploited the results of round 8 of the latest WHO testing programme, as presented in Table 4 [30]. Results were available for seven of the total number of RDTs evaluated from the included studies (Table 2). None of these RDTs meet all WHO performance criteria in terms of PDS and positive rate. However, they were all efficient in terms of invalid rate (i.e., <5%) and FP rate (i.e., <10%), except for the “Parascreen Device Pan/Pf” RDT (FP rate = 50.6%) (Table 4).

## 4. Discussion

In this review, the performances of malaria RDTs were evaluated from studies carried out in India, since their implementation in malaria endemic countries in the 1990s.

The pooled estimates of sensitivity and specificity were below 100%, meaning that RDTs misclassified some individuals (i.e., FN and FP). FP is particularly seen in RDTs targeting PfHRP2 protein, as this protein is detectable in the bloodstream for many weeks following successful treatment of infections [4]. In addition, we found that the Se values of PfHRP2-based RDTs decreased over these three decades. Using a bespoke Bayesian survival model, Dalrymple et al. estimated that 50% and 5% of PfHRP2-based RDTs were still positive 15- and 36-days post-treatment, respectively [31]. Several causes can explain FN, viz., low parasitemia, poor handling of the RDTs, poor storage of the RDT, prozone effect (i.e., FN or false-low results in antibody-antigen reactions, due to an excess of either antibodies or antigens), and recently deletions in the *pfhrp2* gene [11,32,33].

The RDTs’ performance parameters were evaluated using LM and PCR as the gold standard. These performance parameters were lower in studies having used PCR compared with studies having used LM. Molecular methods are more sensitive than LM [12], and thus some samples LM-negative and/or RDT-negative can be revealed as positive by using molecular methods. These types of infections are termed sub-microscopic infections [34]. The extent of these infections varies according to geographical areas and level of endemicity, and sub-microscopic infections were showed to play a great role in malaria transmission and morbidity [35,36].

Only one study evaluated the RDT performances against *P. malariae*, while none of the studies focused on the other non-falciparum/vivax species (i.e., *P. ovale* and *P. knowlesi*). *P. knowlesi* has been reported from four states till now in India though *P. ovale* is rare in the country [37]. *Plasmodium malariae* has been reported as the most frequent non-falciparum/vivax species across India, and are mainly seen as co-infection cases with *P. falciparum* [37,38,39,40]. Performances of RDTs against *P. ovale* and *P. malariae* has been shown to be suboptimal [41]. It is also worth addressing the detection of these species by developing specific RDTs, as some reports of complications due to these species have started to accrue in some areas [42,43,44].

Gametocytes are not responsible for the disease, but they are involved in malaria transmission. Thus, the detection of gametocyte carriers is crucial to efficiently reduce the transmission reservoir of *Plasmodium* parasites, and thus reduce the malaria burden. Contradictory findings were reported on the reactivity of RDTs to gametocytes, and these concerned PfHRP2-based RDTs only. PfHRP2 is produced at varying levels, depending on the development stage (highly produced by ring stage, moderately by trophozoites, lowly by immature gametocytes and not at all by mature gametocytes) [14,45]. Thus, PfHRP2-based RDTs can still give positive results against immature gametocytes and negative against mature gametocytes. All else being equal, it is likely that these discrepancies are due to differences in maturity of gametocytes in patients from these different studies. Besides, PfHRP2-based RDTs can give a positive result in infections with only mature gametocytes if HRP2 is still circulating at a blood-detectable level, but they will probably give negative results in cases of infection with only mature gametocytes and absence/non-detectable level of HRP2.

The WHO, for the first time, has recently evaluated the performances of some RDTs against *pfhrp2*-negative *P. falciparum* samples [30]. The results were available for seven of all RDTs identified in our review, and none of them met the WHO-defined performance criteria (Table 4). Actually, just two RDTs, viz., “CareStart^™^ Malaria PAN (pLDH) Ag” and “careUS^™^ Malaria PAN (pLDH) Ag”, satisfy the WHO performance criteria for detection of *pfhrp2*-deleted parasites [30]. The WHO recommends not to use exclusively HRP2-detecting RDTs in areas where FN result rates, due to *pfhrp2* deletions, ≥5% [46]. There are rising reports on the presence of *P. falciparum* isolates with *pfhrp2* deletions in India, which constitute an undetected reservoir for malaria transmission [47,48,49,50]. These studies were not designed for estimating of *pfhrp2/3* deletions among symptomatic patients with RDT-negative results [46]. Thus, it is would be premature to think about changing HRP2-detecting RDTs in the country. However, it would also be interesting to conduct such types of studies to know whether HRP2-detecting RDTs are still needed in India. In parallel, it would be interesting to develop RDTs targeting other antigens such as glutamate dehydrogenase, recently proposed and shown as a good candidate [51].

## 5. Limitations of this Review

The present review has some limitations. First, a high level of heterogeneity was found between studies. Second, the existence of a selection bias is probable as only studies written in the English language were included in the analysis. Third, we tested some variables in subgroup analysis; however, confounding variables such as the level of endemicity (information that was given only in the 13 included studies) and the level of parasitemia were not investigated as an additional source of between-study heterogeneity. These variables were not specified in most of the studies included in the review.

## 6. Conclusions

In conclusion, RDTs are still performants for the detection of malaria parasites in India. However, they are subject to result variability due to many factors (e.g., geographical area, antigen targeted, presence of gametocytes). This study also outlines the need for development of RDTs for the detection of minor malaria species circulating in the country (i.e., *P. ovale, P. malariae* and *P. knowlesi*) to reach malaria elimination objectives. It is also crucial to target individuals carrying only mature gametocytes involved in malaria transmission. Finally, in a current context of deletions in the *pfhrp2* gene hindering the accuracy of PfHRP2-based RDTs, it would be interesting to conduct studies to determine the prevalence of *pfhrp2/3* deletions among patients with RDT-negative results. Finally, it is also important to identify new malarial antigens with a potential to be used as markers for malaria diagnosis in the future.

## Figures and Tables

**Table 1:** Data of interest retrieved from each included study.

Types of Information	Nature of the Information Retrieved
**Information on the Publication**	Name of the first author or of the two authors Year of publication
**Information on study design, population, area**	Study design Indian areas and Level of endemicity of area Year of data collection Population (adult, children and pregnant women) Clinical status of patients (febrile, asymptomatic) Type of malaria
**Diagnostic**	Diagnostic methods used (LM, RDT, molecular methods) Staining molecule used in LM (Giemsa, Leishman, Jaswant Singh–Bhattacharji) Type, antigen, brand and manufacturer’s country of the RDTs Number of RDTs evaluated
**Performance parameters**	Total number of individuals tested with different methods Total number of patients positive with gold standard and RDT Total number of *P. vivax* infections Number of mixed infections Number of false negative (FN), false positive (FP), true negative (TN) and true positive (TP) for diagnosis of *P. falciparum*, *P. vivax* and non-falciparum species.

**Table 2:** Details of RDTs from the included studies based on rounds 5–8 of RDT testing programme [30].

Brand	*n*	Manufacturer	Antigen Targeted	Categorisation as per WHO	PDS (%) at 200 Parasites/µL (Rounds 5–8) for Pf^a^ and Pv^b^	FP and Invalid Result Rates	WHO Performance Criteria
ParaCheck Pf^®^	12	Orchid, Biomedical Systems, Goa, India	HRP2	Pf only	94.0^a^ and NA^b^	NA and 0%	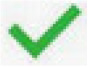
ParaSight F^®^	10	Becton Dickonson, Cockeys ville, MD, USA	HRP2	Pf only	-	-	-
ICT Malaria Pf^™^	10	ICT Diagnostics, Brooksvale, NSW, USA	HRP2	Pf only	-	-	-
ParaHIT^®^-f	9	ARKRAY Healthcare Pvt. Ltd., Surat, India	HRP2	Pf only	77.0^a^ and NA^b^	NA	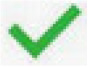
DiaMed OptiMAL IT^®^	6	DiaMed AG, Cressier, Switzerland	PfLDH + Pan-LDH	Pf/Pan	-	-	-
ICT Malaria Pf/Pv^™^	4	ICT Diagnostics, Brooksvale, NSW, USA	Panmalarial Ag + HRP2	Pf and Pv	94.0^a^ and NA^b^	NA	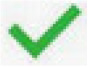
ParaHIT^®^ Total	4	ARKRAY Healthcare Pvt. Ltd., Mumbai, India	Aldolase + Pan-LDH + HRP2	Pf/Pan	-	-	-
SD Bioline Pf/Pan^®^	4	SD Standard Diagnostics, Inc., South Korea	PfLDH + Pan-LDH	Pf/Pan	94.0^a^ and 91.4^b^	-	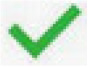
Determine^™^ Malaria Pf	3	Abbot Laboratories, Tokyo, Japan	HRP2	Pf only	-	-	-
Parascreen Device Pan/Pf^®^	3	Zephyr Biomedical, Verna, Goa, India	Pan-LDH + HRP2	Pf/Pan	91.0^a^ and 91.4^b^	0% and 0%	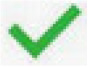
FalciVax^™^ (Pf/Pv)	3	Zephyr Biomedical, Verna, Goa, India	PvLDH + HRP2	Pf and Pv/Pvom	95.0^a^ and 100^b^	0.5% and 0%	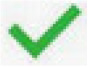
First Response Combo Malaria Ag^®^	3	Premier medical corporation Ltd., India	Pan-LDH + HRP2	Pf/Pan	91.0-95.0^a^ and NA^b^	0% and 0%	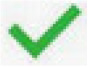
SD Bioline Pf/Pv^®^	3	SD Standard Diagnostics, Inc., South Korea	PfLDH + PvLDH	Pf and Pv	99.0^a^ and 97.1^b^	0% and 0%	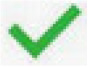
Advantage Mal Card^™^	2	J. Mitra & Co. Pvt. Ltd., Rajasthan, India	PfLDH + Pan-LDH	Pf/Pan	30.0^a^ and 94.3^b^	0.4% and 0%	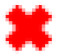
Alere^™^ Trueline Malaria Ag Pf/Pan	1	Alere Medical Pvt. Ltd., India	Pan-LDH + HRP2	Pf/Pan	85.0^a^ and 91.4^b^	0% and 0%	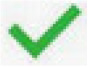
DiaMed OptiMAL^®^	2	Flow Inc., Portland, OR, USA	Pan-LDH	Pan only	-	-	-
SD Bioline Pf^®^	2	SD Standard Diagnostics, Inc., South Korea	HRP2	Pf only	94.0^a^ and NA^b^	NA and 0%	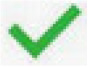
Malaria Pf (HRPII)/PV (PLDH) Antigen Detection Test Device^™^	1	GENOMIX Molecular Diagnostics Pvt. Ltd., Hyderabad, Andhra Pradesh, India	PvLDH + HRP2	Pf and Pv/Pvom	85.0^a^ and 74.3^b^	NA	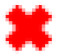
Malarigen^™^ Pf/Pv test	1	Aspen Laboratories, India	Aldolase + Pan-LDH	NA	-	-	-
Malarigen^™^ Pf/Pv test	1	Aspen Laboratories, India	Pan-LDH + HRP2	NA	-	-	-
Malarigen^™^ Pan test	1	Aspen Laboratories, India	Pan-LDH	NA	-	-	-
Malascan Device Pf/Pan^®^	1	TulipScan Diagnostics, India	Aldolase + HRP2	Pf/Pan	-	-	-
NecVIPARUM^™^ One step Pf/Pv	1	Nectar Life Science Ltd., Chandigarh, India	PvLDH + HRP2	Pf and Pv/Pvom	88.0^a^ and 91.4^b^	0% and 4.5%	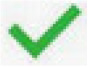
New^™^ Pf-1 mini	1	Monozyme India Ltd., Secundradad, India	HRP2	Pf only	-	-	-
DiaMed OptiMAL^®^ 48	1	DiaMed AG, Cressier, Switzerland	Pan-LDH	Pan only	-	-	-
Standard Q^™^ Pf/Pv	1	SD Biosensor healthcare Pvt. Ltd., India	PvLDH + HRP2	Pf and Pv/Pvom	85.0^a^ and 100^b^	0% and 0.5 %	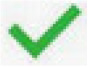
Not specified	11	-	-	-	-	-	-
Total	101^§^						

PDS: Panel detection score; Pf = *P. falciparum*, Pv = *P. vivax*, Pvom = *P. vivax*, *P. ovale* and *P. malariae*. FP: False positive; HRP2 = Histidine rich protein 2, LDH = Lactate dehydrogenase, NA = Not applicable; WHO = World Health Organisation. RDTs meeting the following WHO performance criteria are recommended for procurement: PDS ≥ 75% for the detection of *P. falciparum* and *P. vivax* at 200 parasites/µL in all malaria settings, FP rate < 10% and invalid rate < 5% [30]. ^a^PDS (%) at 200 parasites/µL for *P. falciparum*; ^b^PDS (%) at 200 parasites/µL for *P. vivax*; The green check means that the RDT meets, where applicable, the WHO performance criteria; The red cross mark means that the RDT does not meet at least one of the WHO performance criteria(-): the PDS results are missing due to many reasons (results have been removed from the summary WHO result listings, manufacturer did not submit its product for evaluation, RDT is no longer manufactured).§: The total is more than the total number of the included studies (*n* = 75) as some studies evaluated more than one RDT.

**Table 3:** Reactivity of RDT according to gametocyte carriage and level of parasitemia.

	RDT	Authors’ Findings	References as Seen in Table S4
**Gametocyte detection *versus* RDT positivity**	ICT Malaria Pf^™^	Presence or absence of gametocytes was not related to RDT positivity	Valecha et al._1998
	ICT Malaria Pf^™^	Presence or absence of gametocytes was not related to RDT positivity	Ghosh et al._2000
	ParaCheck Pf^®^	No correlation between presence of falciparum gametocytes and RDT positivity	Ghosh et al._2002
	ParaCheck Pf^®^	Three individuals infected only with falciparum gametocytes were all RDT positive	Singh et al._2002
	ParaCheck Pf^®^	Two individuals infected only with falciparum gametocytes were all RDT negative	Arora et al._2003a
	ParaSight F^®^	RDT failed to detect one individual with falciparum gametocytes only	Arora et al._2003b
	DiaMed OptiMAL^®^	Two individuals infected only with falciparum gametocytes were all positive with the RDT	Singh et al._2003
	ParaCheck Pf^®^	*P. falciparum* gametocytes not detected or weakly detected by the RDT in all carriers	Gokhale et al._2004
	ParaHit^®^-f	14 individuals infected with falciparum gametocytes were RDT positive	Singh et al._2005a
	ParaCheck Pf^®^	Three only falciparum gametocytes -infected individuals were all RDT positive	Bhat Sandhya et al._2012
	Advantage mal Card^™^	On day 8, falciparum gametocytes were detected by the RDT in all 9 patients	Kocharekar et al._2014
**Parasitemia *versus* Intensity band of RDT**	ICT Malaria Pf^™^	No correlation found	Valecha et al._1998
	ICT Malaria Pf^™^	No correlation found	Ghosh et al._2000
	ParaCheck Pf^®^	Positive correlation for development stage (Ring only, and Ring + gametocyte)	Ghosh et al._2002

**Table 4:** Performances of RDTs against *pfhrp2*-negative *P. falciparum* samples based on results of round 8 of the WHO testing programme [30].

		WHO Performance Indicators^&^				
RDTs	Antigen Targeted	PDS (%)	Positivity Rate (%)	FP Rate (%)	Invalid Rate (%)	Overall Evaluation
SD Bioline Pf/Pan	PfLDH + Pan-LDH	32.5	68.1	NA	0.0	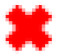
ParaCheck^®^ F Pf	HRP2	15.0	40.5	NA	0.0	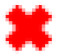
Malarigen Pf/Pv	HRP2 + Pan-LDH	32.5	43.1	0.0	0.0	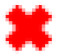
FalciVax (Pf/Pv)	HRP2 + Pv-LDH	0.0	3.1	0.0	0.0	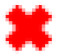
First Response^®^ Combo Malaria Ag	HRP2 + Pan-LDH	12.5	23.1	0.0	0.0	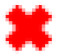
NecVIPARUM One step Pf/Pv	HRP2 + Pv-LDH	32.5	45.9	0.6	0.6	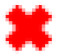
Parascreen Device Pan/Pf	HRP2 + Pan-LDH	0.0	0.6	50.6	0.0	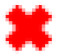

HRP2: Histidine rich protein 2; LDH: Lactate dehydrogenase; PDS: Panel detection score; FP: False positive; NA: Not applicable; Pf: *P. falciparum*; Pv: *P. vivax;* WHO: World Health Organisation. ^&^RDTs meeting the following WHO performance criteria are recommended for procurement: PDS ≥ 75% for the detection of *P. falciparum* and *P. vivax* at 200 parasites/µL in all malaria settings, Positive rate ≥ 75%, FP rate < 10% and invalid rate < 5% [30]. The red cross mark means that the RDT failed to meet all the WHO performance criteria altogether.

## Data Availability

The data presented in this study are available on request from the corresponding author.

## Supplementary Materials

The following are available online at https://www.mdpi.com/2075-4418/11/4/590/s1. Figure S1: PRISMA flow diagram, Table S2: PRISMA checklist, Table S3: List of studies excluded in the review with reasons for exclusion, Table S4: List of studies included in the review, Table S5: Results on quality assessment of the included studies, Figure S6: Forest and funnel plots of pooled values of diagnostic performance parameters across all included studies, Table S7: Results of subgroup analysis on performances of RDTs in the detection of at least *P. falciparum* and/or *P. vivax*, *P. falciparum* only, and *P. vivax,* Figure S8: Results of sensitivity analysis on performances of RDTs in the detection of at least *P. falciparum* and/or *P. vivax*, *P. falciparum* only, and *P. vivax*.

## Abbreviations

95% CI	Confidence interval at 95%
DOR	Diagnostic odd ratio
FP	False positive
FN	False negative
JBI	Joanna Briggs Institute
LDH	Lactate dehydrogenase
LM	Light microscopy
NLR	Negative likelihood ratio
PDS	Panel detection score
PfHRP2	*P. falciparum* histidine rich protein 2
PLR	Positive likelihood ratio
PRISMA	Preferred Reporting Items for Systematic Reviews and Meta-analyses
RDTs	Rapid diagnostic tests
Se	Sensitivity
SEA	South East Asia
sSA	sub-Saharan Africa
SR-MA	Systematic review and meta-analysis
Sp	Specificity
TP	True positive
TN	True negative
WHO	World Health Organization
