# Supplementary material for: Field Performances of Rapid Diagnostic Tests Detecting Human Plasmodium Species: A Systematic Review and Meta-Analysis in India, 1990–2020
Source: Diagnostics (Basel). 2021 Mar 25;11(4):590. doi: 10.3390/diagnostics11040590 (PMC8064471; doi:10.3390/diagnostics11040590)
Supplement: Supplementary file 1 [file diagnostics-11-00590-s001.zip › Supplementary file 3.doc]

**List of studies excluded from the systematic review**

| **Studies** | **Title** | **Reasons for exclusion** |
| --- | --- | --- |
| **Singh and Shukla_2002** | Short report: Field evaluation of post-treatment sensitivity for monitoring parasite clearance of Plasmodium falciparum malaria by use of the Determine Malaria pf test in central India. | RDT was used to evaluate the efficacy of a malaria treatment |
| **Gurung et al_2010** | Evaluation of Falcivax against Quantitative Buffy Coat (QBC) for the Diagnosis of Malaria | PBF/Molecular methods were not used as gold standard |
| **Jayakumar et al_2011** | Correlation of Quantitative Buffy Coat, Blood smear and Antigen detection in diagnosing Malarial Infection | PBF/Molecular methods were not used as gold standard |
| **Mohan_2014** | An innovative test for rapid diagnosis of malaria | News |
| **Verma et al_2015a** | Novel monoclonal antibody against truncated C terminal region of Histidine Rich Protein2 (PfHRP2) and its utility for the specific diagnosis of malaria caused by Plasmodium falciparum | Experimental study |
| **Verma et al_2015b** | Diagnostic potential of monoclonal antibodies developed against C-terminal polypeptide of P. falciparum Histidine Rich Protein2 (PfHRP2) in malaria infected patients from India. | Experimental study |
| **Morch et al_2017** | Acute undifferentiated fever in India: a multicentre study of aetiology and diagnostic accuracy | RDT used but not evaluated |
| **Samal et al_2017** | The sensitivity and specificity of a urine based Rapid Diagnostic Test for the diagnosis of Plasmodium falciparum in a malaria endemic area in Odisha, India | RDT detecting malaria parasites in urine samples |
| **Saha et al_2017** | Diagnostic efficacy of microscopy, rapid diagnostic test and polymerase chain reaction for malaria using bayesian latent class analysis | RDT performances were used by Bayesian mathematical model |
| **Malledapi et al_2019** | Selecting better diagnostic kits for diagnosis of malarial parasites at point of care | Experimental study |
| **Mahale et al_2019** | Assessment of efficacy of palm polymerase chain reaction with microscopy, rapid diagnostic test and conventional polymerase chain reaction for diagnosis of malaria | RDT used but not evaluated |
| **Other studies** |  | Studies conducted outside India (n = 316), Reviews (n = 25), Epidemiological studies that used RDT as diagnostic tool (n = 23), RDT used to target other infectious diseases (n = 19), Irrelevant (n = 4,519) |
