# Supplementary material for: Field Performances of Rapid Diagnostic Tests Detecting Human Plasmodium Species: A Systematic Review and Meta-Analysis in India, 1990–2020
Source: Diagnostics (Basel). 2021 Mar 25;11(4):590. doi: 10.3390/diagnostics11040590 (PMC8064471; doi:10.3390/diagnostics11040590)
Supplement: Supplementary file 1 [file diagnostics-11-00590-s001.zip › Supplementary file 6.doc]

**Additional file 6**: Forest and funnel plots of pooled values of diagnostic performance parameters across all included studies

I. **Performances of RDTs for detection of *Plasmodium spp* parasites**
1. **Forest plots**
   a. **Sensitivity**

b. **Specificity**

c. **Positive likelihood ratio**

d. **Negative likelihood ratio**

e. **DOR**

2. **Funnel plots**
   a. **Sensitivity**
   b. **Specificity**
   c. **Positive likelihood ratio**
   d. **Negative likelihood ratio**
   e. **DOR**
II. **Performances of RDTs for detection of *Plasmodium falciparum***
1. **Forest plots**
   a. **Sensitivity**

b. **Specificity**

c. **Positive likelihood ratio**

d. **Negative likelihood ratio**

e. **DOR**

III. **Performances of RDTs for detection of *Plasmodium vivax***
1. **Forest plots**
   a. **Sensitivity**

b. **Specificity**

c. **Positive likelihood ratio**

d. **Negative likelihood ratio**

e. **DOR**

2. **Funnel plots**
   a. **Sensitivity**
   b. **Specificity**
   c. **Positive likelihood ratio**
   d. **Negative likelihood ratio**
   e. **DOR**
