# Supplementary material for: Field Performances of Rapid Diagnostic Tests Detecting Human Plasmodium Species: A Systematic Review and Meta-Analysis in India, 1990–2020
Source: Diagnostics (Basel). 2021 Mar 25;11(4):590. doi: 10.3390/diagnostics11040590 (PMC8064471; doi:10.3390/diagnostics11040590)
Supplement: Supplementary file 1 [file diagnostics-11-00590-s001.zip › Supplementary file 8.doc]

Results of sensitivity analysis (leave-one-out analysis) on performances of RDTs in the detection of at least *P. falciparum* and/or *P. vivax*, *P. falciparum* only, and *P. vivax*

I. **Performances of RDTs for detection of *Plasmodium spp* parasites**
   a. **Sensitivity**

b. **Specificity**

c. **Positive likelihood ratio**

d. **Negative likelihood ratio**

e. **DOR**

II. **Performances of RDTs for detection of *Plasmodium falciparum***
    a. **Sensitivity**

b. **Specificity**

c. **Positive likelihood ratio**

d. **Negative likelihood ratio**

e. **DOR**

III. **Performances of RDTs for detection of *Plasmodium vivax***
a. **Sensitivity**

b. **Specificity**

c. **Positive likelihood ratio**

d. **Negative likelihood ratio**

e. **DOR**
