# Supplementary material for: Field Performances of Rapid Diagnostic Tests Detecting Human Plasmodium Species: A Systematic Review and Meta-Analysis in India, 1990–2020
Source: Diagnostics (Basel). 2021 Mar 25;11(4):590. doi: 10.3390/diagnostics11040590 (PMC8064471; doi:10.3390/diagnostics11040590)
Supplement: Supplementary file 1 [file diagnostics-11-00590-s001.zip › Supplementary file 1.doc]

**PRISMA 2009 Flow Diagram**

**Records identified through database searching**

(n =	)

**Screening**

**Included**

**Eligibility**

**Identification**

**Additional records identified through hand searching, Google and Google scholar**

(n = )

**Records after duplicates removed**

(n =	)

**Records screened**

(n = )

**Records excluded**

**Full-text articles assessed for eligibility**

(n =	)

**Articles excluded**

**Studies included in qualitative synthesis**

(n =	)

*From:* Moher D, Liberati A, Tetzlaff J, Altman DG, The PRISMA Group (2009). *P*referred *R*eporting *I*tems for *S*ystematic Reviews and *M*eta-*A*nalyses: The PRISMA Statement. PLoS Med 6(7): e1000097. doi:10.1371/journal.pmed1000097

**For more information, visit** [**www.prisma-statement.org**](http://www.consort-statement.org/)**.**
